# Preliminary Study on the Antibacterial Activities and Antibacterial Guided Fractionation of Some Common Medicinal Plants Practices in Itum Bahal, Kathmandu Valley of Nepal

**DOI:** 10.1155/2023/7398866

**Published:** 2023-09-21

**Authors:** Ravin Bhandari, Dhakaraj Pant, Kamal Singh Kathayat, Ramesh Bhattarai, Himal Barakoti, Jitendra Pandey, Nirmala Jamarkatel-Pandit

**Affiliations:** ^1^Department of Pharmacy, School of Medicine, Karnali Academy of Health Sciences, Jumla 21200, Nepal; ^2^Department of Medical Laboratory Science, School of Health and Allied Sciences, Pokhara University, Kaski 33700, Nepal; ^3^School of Public Health, Karnali Academy of Health Sciences, Jumla 21200, Nepal; ^4^School of Medicine, Karnali Academy of Health Sciences, Jumla 21200, Nepal; ^5^Department of Pharmacy, Purbanchal University, Biratnagar 56613, Nepal; ^6^Department of Chemistry, University of Hawai'i at Manoa, 2545 McCarthy Mall, Honolulu, HI 96822, USA; ^7^Department of Pharmaceutical Science, School of Health and Allied Science, Pokhara University, Kaski 33700, Nepal

## Abstract

The aim of this study was to evaluate the antibacterial activities of selected medicinal plant practices by a traditional healer of the Newar community in Itum Bahal, Kathmandu, Nepal. The antibacterial activities of the methanolic extract (1 mg/disc) of fifteen medicinal plants were screened against two Gram-positive bacteria (*Staphylococcus aureus* ATCC 25923 and *Bacillus subtilis* ATCC 6633) and two Gram-negative bacteria (*Escherichia coli* ATCC 25922 and *Salmonella typhi* CCM 5445) using the disc diffusion method. Minimum inhibitory concentration (MIC) and minimum bactericidal concentration (MBC) were calculated for the different fractions (hexane, chloroform, ethyl acetate, acetonitrile, and acetone) of the plants having a significant antimicrobial effect. Only *Quercus infectoria* G. Olivier (galls) and *Mallotus repandus* (Willd.) Müll.Arg. (seeds) exhibited prominent antibacterial effects. The acetone fraction from *Q. infectoria* had the strongest antibacterial effect, with a 41.00 mm inhibition zone against *S. aureus*. In contrast, the ethyl acetate fraction in *M. repandus* exhibited the highest efficacy, producing a 29.00 mm inhibition zone against *S. typhi*. In a similar manner, in the case of *Q. infectoria*, the acetoe fraction depicted the lowest MIC (0.19 mg/mL) and MBC (0.98 mg/mL) values against *S. aureus*, whereas the ethyl acetate fraction of *M. repandus* was most significant, showing the lowest MIC and MBC of 0.25 and 0.53 mg/mL, respectively, against *S. typhi.* This study suggested that the acetone extract of *Q. infectoria* galls can be used as a potential source against Gram-positive bacteria, whereas the ethyl acetate extract of *M. repandus* seeds could serve as a useful source to inhibit Gram-negative bacteria. Furthermore, extensive scientific investigation is mandatory to ensure the proper use of folk medicines.

## 1. Introduction

Conventional utilization of plants for remedial purposes anticipates a basis for the employment of particular plants for specific medicinal conditions [1, 2]. Herbal medicine carries on furnishing health treatment for over 80% of the total population in the world, largely in developing countries [3–6]. Therapeutic properties of plant materials have been documented and substantiated experimentally in diverse cultures since ancient times [7, 8]. Early civilizations such as Indian, Chinese, and the Middle East have recorded the medicinal values of thousands of plants. In recent times, numerous industries beyond the realm of food and medicine have shown increasing interest in exploring the potential of medicinal plants. Extensive research is being conducted to investigate the properties of these plants in various domains, focusing on their active ingredients for applications such as antioxidants, phenolic content, anticancer properties, DNA protection, antiproliferative effects, and antimicrobial activities. These studies aim at uncovering the valuable properties of medicinal plants that extend beyond their traditional therapeutic uses, opening up new possibilities and applications in diverse sectors [9–12]. Nepal is a prosperous country in the biodiversity of medicinal plants. Approximately, 7000 species of medicinally significant plants have been discovered all around the world. In Nepal, 6,653 species of angiosperm plants have been documented. Among these, about 25%–50% are ethnomedicinally useful. Around 1,792–2,331 beneficial aromatic and medicinal plants are recorded in catalogs. Woefully, just a few of them are being practiced for their therapeutic value [13–16]. Herbal medicine represents one of the most important fields of traditional medicine in Nepal especially in rural areas [17–19]. A large number of traditional healers have been playing a pivotal role in narrowing the gap between rural and urban Nepal as they are more accessible than doctors having training in Western medicine [20, 21]. The present paper deals with the investigation of the antibacterial potency of 15 different medicinal plants that have been used by the Newar community traditional healers of Itum Bahal, Kathmandu, Nepal, for the treatment of different kinds of diseases.

Even with substantial advancement in scientific comprehension and medical technology, diseases caused by microbial infection persist as a prime cause of worldwide mortality and morbidity [22, 23]. Although deaths from bacterial infection have dropped in the developed countries, it is still a major cause of death in the developing world. According to WHO, 1.9 million children died worldwide due to respiratory infections with 70% of these deaths occurring in Africa and Asia in 2000 [24]. In developed countries, food poisoning caused by the virulent strain of *E. coli* is still the major cause of death every year. The exploration of antibiotics has suppressed the microbial infection that once devastated the humankind. Unfortunately, the irrational consumption of antimicrobial drugs for the treatment of infectious diseases has resulted in the development of multidrug-resistant microorganism strains [25–28]. Unavailability of everlasting effective drugs and the exorbitant price of new generation antibiotics have resulted in an increased rate of mortality and morbidity accompanying the development of uncertainty regarding the prospects of antimicrobial drug use [29]. This catastrophic circumstance has impelled us to investigate more successful antimicrobial agents utilizing plant material so that it will provide active therapeutic ingredients as well as lead molecules for the synthesis of perfect novel drugs [30].

## 2. Materials and Methods

### 2.1. Plant Material

**Figure 1:**
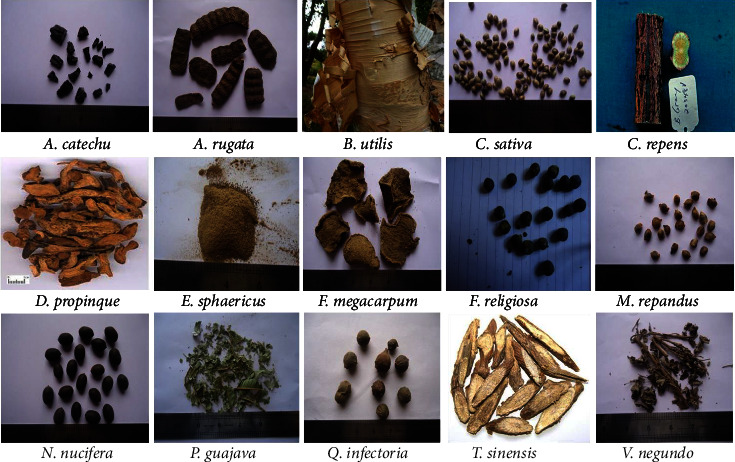
Photographs of used parts of fifteen different medicinal plants from Itum Bahal Kathmandu, Nepal.

Dried raw material (not less than 500 g) of each selected plant variety (Table 1 and Figure 1) was collected from the Itum Bahal market of Nepal where a traditional healer practices all the collected plants for several cases of upper respiratory tract and lower urinary tract infection. The scientific name of all the materials was identified from their local name with the help of a botanist (Homnath Pathak from Prithvi Narayan Campus, Nepal) and also compared with the literature. The voucher specimens were carefully labeled and safely stored in the herbarium unit of Pokhara University, Kaski, Nepal. The corresponding voucher numbers for all the specimens are given in Table 1.

### 2.2. Preparation of Extracts

Cleaned shade-dried plant materials were comminuted into fine powders. Each dried sample (100 g) was extracted by double maceration using 500 mL methanol (MeOH) at room temperature for 48 hours. The MeOH extracts were evaporated to dryness using a rotatory vacuum evaporator at 40°C and stored in the refrigerator at a temperature of 4°C until use.

### 2.3. Antibacterial Bioassay-Guided Fractionation

**Figure 2:**
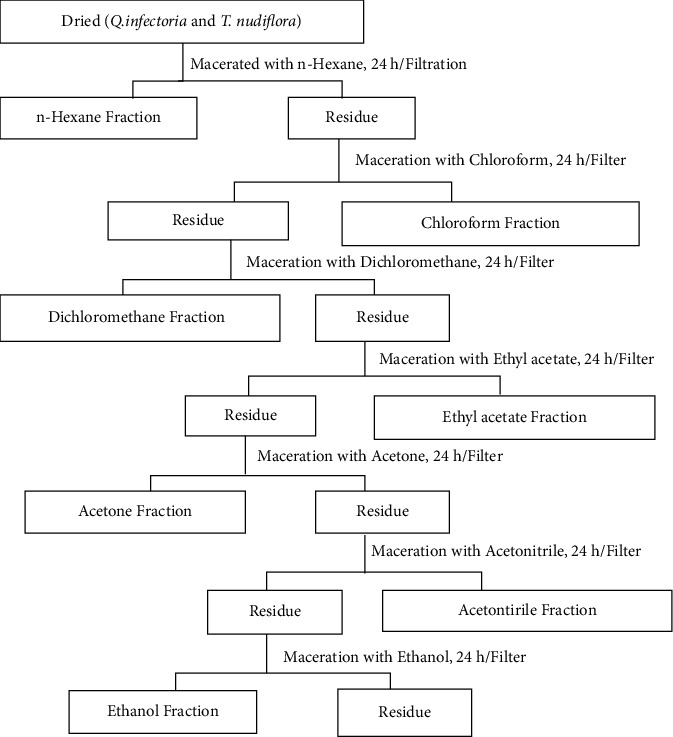
Schematic representation for antibacterial bioassay guided fractionation.

Initially, methanolic extracts of all 15 samples were screened for the zone of inhibition shown by each extract. However, only *Q. infectoria* and *M. repandus* methanolic extract showed the measurable zone of inhibition. Hence, the methanolic extract of only these two plants were further partitioned with hexane, chloroform, dichloromethane, ethyl acetate (EtOAc), acetone, acetonitrile, and ethanol (EtOH) sequentially as shown in Figure 2. Each fraction thus obtained, including the final EtOH fraction, was evaporated to dryness and subjected to antibacterial activity.

### 2.4. Determination of Antibacterial Activity

#### 2.4.1. Collection of Microorganisms

Four different microbial strains, *E. coli* ATCC 25922, *B. subtilis* ATCC 6633, *S. typhi* CCM 5445, and *S. aureus* ATCC 25923, were purchased from Teku, Kathmandu, Nepal.

#### 2.4.2. Preservation, Establishment, and Optimization of Stock Microbial Cultures

The stock microbial cultures were preserved on a solid nutrient agar medium at 40°C. Subcultures were newly prepared in liquid medium (nutrient broth) and incubated at 37°C for 24 hours before use. Standard suspensions of each test microorganism were made by comparing with the turbidity of 0.5 McFarland solution standards. A UV spectrometer is used to adjust turbidity to match the McFarland standard (absorbance 0.1 at 600 nm) to get about 1 × 10^8^ cells/mL to obtain the requisite working suspension (1–5 CFU/mL) [16, 26].

#### 2.4.3. Solvent, Test Sample Solution, and Standard Drugs

25% v/v of dimethyl sulfoxide (DMSO) was used as a solvent system. A stock solution of a concentration of 100 mg/mL was prepared for each extract. A sterile paper disc (6 mm diameter) was impregnated with 10 *μ*L of stock and allowed to dry at 37°C for 24 hours to give a final amount of 1 mg plant extract per disc. Amoxicillin 30 *μ*g/disc and norfloxacin 10 *µ*g/disc, obtained from HiMedia Laboratories Private Limited, India, were used as positive controls [31, 32].

#### 2.4.4. Antimicrobial Screening by the Disc Diffusion Method

Preliminary antimicrobial screening for the methanolic extract of 15 different plants was carried out according to the disc diffusion method described in a previous study. The Mueller–Hinton agar (MHA) medium was prepared and sterilized at 121°C for 20 minutes in the autoclave. About 25 mL sterilized media was poured into each 12 cm sterile Petri dishes under aseptic conditions and allowed to settle. The bacterial suspension was lawn cultured over the agar medium using a sterile glass-L rod. The extract disc was gently placed at equidistant on top of the agar layer. 25% DMSO containing disc was used as a negative control. The reference antibiotics disc of amoxicillin (30 *μ*g/disc) and norfloxacin (10 *μ*g) as positive controls were also placed at equidistant, on top of the agar layer. Plant extracts disc (1 mg/disc) and reference drugs were allowed to diffuse for 1 hour into the plates and then incubated at 37°C for 24 hours in an inverted position. The results were obtained by measuring the zone of growth inhibition (mm) surrounding the discs. Each attempt was performed in triplicates [26, 33].

#### 2.4.5. Determination Minimum Inhibitory Concentration (MIC)

Seven vials were properly sterilized and labeled for the examination of individual extracts. Each vial was loaded with 750 *µ*L of sterile Mueller–Hinton broth (MHB). In order to make the samples, a concentrated solution of 40 mg/mL was prepared using DMSO. The initial concentrated solution underwent a series of dilutions to produce seven distinct concentrations of the extract, spanning from 40 mg/mL down to 0.625 mg/mL. This dilution process involved dissolving the concentrated solution in a 1 : 1 blend of DMSO and water. Following this, 250 *µ*L of the resulting sample solution was meticulously transferred into the corresponding vial containing 750 *µ*L of MHB. Consequently, the concentration of the sample solution in every vial varied from 10 mg/mL to 0.1562 mg/mL. Bacterial cultures with an initial concentration of around 1 × 10^5^ CFU/mL were introduced into all vials, including solvent blanks, negative controls, and positive controls. Subsequently, the vials were tightly sealed and placed in an incubator set at 37°C for a duration of 24 hours. After this incubation period, the MIC values were assessed. MIC denotes the lowest concentration of the plant sample capable of halting the observable growth of the bacterial culture. The presence of viable bacterial cells was verified through an assay utilizing 3(4,5 dimethylthiazol-2-yl)-2-5-diphenyl tetrazolium bromide (MTT). This process entailed subjecting the samples to further 2-hour incubation at 37°C and visually examining the development of formazan, a byproduct that signifies the presence of viable cells [17, 34, 35].

#### 2.4.6. Determination of Minimum Bactericidal Concentration (MBC)

To ascertain the MBC, refrigerated MHA plates were placed in an incubator at 37°C for 45 minutes, after which they were moved to a sanitized laminar airflow (LAF) hood. Following this, aliquots from each diluted test tube, prepared using the same method as in the MIC assessment, were transferred onto MHA plates. These subcultured plates were subsequently placed in an incubator set at 37°C for a period of 24 hours. The MBC was characterized as the minimum concentration of the plant extract that entirely suppressed bacterial growth on the culture media's surface [16, 33, 34].

#### 2.4.7. Statistical Analysis

All the values and data were expressed as the mean ± SEM. All the experiments were carried out in triplicate (*n* = 3).

## 3. Results

A total of fifteen species of plants belonging to 15 different families (Table 1), every herb popular within traditional medicine of Nepal, were chosen for the antibacterial activity assay against four different microbial strains. This antibacterial potency of all samples was quantitatively confirmed by the absence or presence of an inhibition zone all over the disc loaded with extract. The result confirmed that only *M. repandus* and *Q. infectoria* showed potent antibacterial effects in all tested organisms (Table 2). The other thirteen plant extracts could not show a measurable zone of inhibition against all microorganisms. In this study, *Q. infectoria* was found to be more effective against Gram-positive bacteria than Gram-negative bacteria, while *M. repandus* was more effective against Gram-negative bacteria than Gram-positive bacteria. Between the two Gram-positive bacteria, *S. aureus* was found to be more sensitive than *B. subtilis* to *Q. infectoria* extract, while *B. subtilis* was more susceptible than *S. aureus* to *M. repandus* extract. However, in the case of Gram-negative bacteria, both plant extracts exhibited more sensitivity towards *S. typhi* than *E. coli*. Both plant extracts performed strong growth inhibition of all strains with minimum inhibitory concentration (MIC) values ≤ 2.5 mg/mL and minimum bactericidal concentration (MBC) values ≤ 10 mg/mL, as given in Table 3.

**Figure 3:**
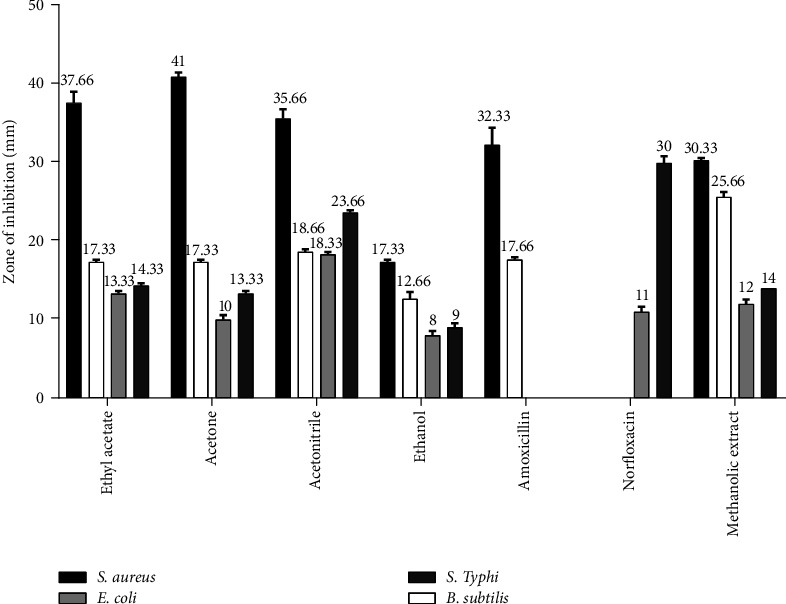
Zone of inhibition (mm) of the *Q. infectoria* extract in different solvent systems compared with standard antibiotics. Note: fraction of *Q. infectoria* (1 mg/disc), amoxicillin (30 *μ*g/disc), and norfloxacin (10 *μ*g/disc).

**Figure 4:**
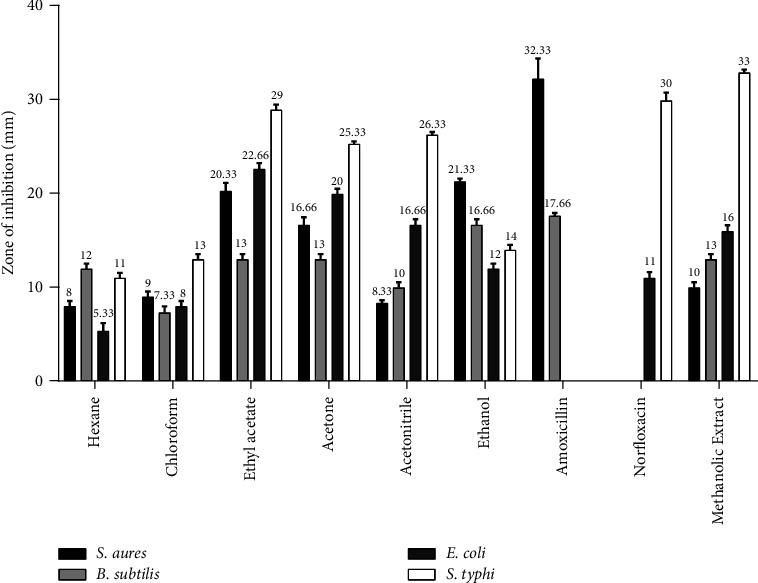
Zone of inhibition (mm) of the *M. repandus* extract in different solvent systems compared with standard antibiotics. Note: fraction of *M. repandus* (1 mg/disc), amoxicillin (30 *μ*g/disc), and norfloxacin (10 *μ*g/disc).

Furthermore, antibacterial assay-guided fractionation of *Q. infectoria* (Figure 3) and *M. repandus* (Figure 4) displayed a variable measure of inhibition zone among different fractions. The acetone fraction of *Q. infectoria* showed the highest inhibition zone for *S. aureus* (ZOI-41 mm), whereas the maximum zone of inhibition against *B. subtilis* (ZOI-18.66 mm), *E. coli* (ZOI-18.33 mm), and *S. typhi* (ZOI-23.66 mm) was performed using its acetonitrile fraction. EtOH extract was reported to be least effective against all strains. As shown in Figure 4, *M. repandus* yielded six fractions, nonpolar fractions such as hexane and chloroform showed lesser activity towards all strains. Among the six fractions, the EtOAc fraction displayed the greatest inhibition zones of 20.33, 13.00, 22.66, and 29.00 mm against *S. aureus, B. subtilis, E. coli,* and *S. typhi*, respectively. Acetone fraction was also significant after EtOAc fraction. The acetonitrile portion was more effective against Gram-negative bacteria than against Gram-positive bacteria. Oppositely, the EtOH fraction revealed greater activity against Gram-positive bacteria with mild activity against Gram-negative bacteria.

According to Table 3, MIC and MBC analyses of *Q. infectoria* revealed that the lowest values of MIC (0.19 mg/mL) and MBC (0.98 mg/mL) were shown by its acetone fraction against Gram-positive bacteria *S. aureus,* whereas, in the case of Gram-negative bacteria, its acetonitrile fraction was found to be more effective with MIC and MBC values of 0.61 mg/mL and 1.31 mg/mL, respectively. Similarly, in the case of *M. repandus,* the lowest value of MIC (0.25 mg/mL) and MBC (0.53 mg/mL) was exhibited by EtOAc fraction against Gram-negative bacteria *S. typhi*. Similarly, in the case of Gram-positive bacteria, EtOH fraction was found to be most prominent with MIC and MBC values of 0.59 mg/mL and 2.35 mg/mL, respectively, against *S. aureus.*

## 4. Discussion

The study of ethnobotanical information about the plants that were used in this study revealed that all the plants are being used for the treatment of the same class of pathogens in different areas of Nepal by different tribes. For example, the juice from the root of *M. repandus* is used to treat bacterial pathogen-associated problems such as stomach complaints such as indigestion, external gouty rheumatism, diarrhea, and dysentery [15].

The preliminary finding obtained from the investigation of the crude methanolic extracts recommended that all plants having ethnomedicinal use may not ensure its therapeutic efficacy. Thus, further investigation is necessary to minimize the irrational use of the medicinal plants. In this study, only *Q. infectoria* and *M. repandus* showed a worthwhile antibacterial activity, wherein the antibacterial activity of *Q. infectoria* MeOH extract showed a greater inhibition zone in Gram-positive bacteria than Gram-negative bacteria. Generally, plant extracts are more active against Gram-positive bacteria than Gram-negative bacteria due to the presence of lipopolysaccharides in the multilayered cell wall composition of Gram-negative strains [31, 35]. It is interesting to note that *M. repandus* MeOH extract showed a greater inhibition zone in Gram-negative bacteria than the Gram-positive bacteria.

The MIC values shown by the MeOH extracts of the *Q. infectoria* galls against *S. aureus, B. subtilis*, *S. typhi,* and *E. coli* were 0.31 mg/mL, 0.62 mg/mL, 1.25 mg/mL, and 2.5 mg/mL, respectively. The lower MIC values of the extract against Gram-positive bacteria, in comparison to Gram-negative bacteria, suggest that the extracts of the *Q. infectoria* galls can suppress Gram-positive bacteria more efficiently [27, 36]. Furthermore, bioassay-guided fractionation of *Q. infectoria* yielded only four fractions, namely, acetone, ethyl acetate, acetonitrile, and ethanol. Among them, the acetone fraction showed the greatest inhibition zone against *S. aureus*, whereas the EtOH fraction had the least inhibition zone against all strains. Acetone and EtOAc fractions had a similar inhibition zone to *B. subtilis*, while the ethyl acetate fraction showed a lesser effect on *S. aureus* than the acetone fraction but greater activity than the other remaining fractions. The EtOAc fraction had a better inhibition zone in Gram-negative bacteria than the acetone fraction. Acetonitrile fraction showed a smaller inhibition zone than acetone and EtOAc fraction to *S. aureus* but formed a higher inhibition zone to *B. subtilis, E. coli,* and *S. typhi*. The MIC values of EtOH and MeOH extract in the study were found to be lower than results of the previous study, against *S. aureus* and *E. coli* [37].

The MBC values for all the fractions were greater than the MIC values against all the bacteria, which advises that the bioactive chemicals in the extract have higher extent of bacteriostatic effect as compared to bactericidal, against all bacteria strains. In contrast, MIC and MBC for acetone extract of *Q. infectoria* galls were reported to be the same in a previous study [27]. By observing the data of zone of inhibition (Table 2 and Figure 3) and MIC and MBC (Table 3), we can conclude that the acetone extract of *Q. infectoria* (gall parts) is most suitable to inhibit the *S. aureus* bacteria, whereas the acetonitrile extract can be the best option to achieve a broad-spectrum activity. Many scientific investigations have asserted that the presence of abundant hydrolysable and condensed tannins in this plant might be responsible for broad-spectrum antibacterial activity as they can stop protein synthesis required for bacterial wall synthesis through the formation of an irreversible complex with proline-rich protein [27, 38, 39].

Bioassay-guided fractionation of *M. repandus* yielded six fractions: hexane, chloroform, acetone, EtOAc, acetonitrile, and EtOH. As shown in Figure 4, the EtOAc fraction exhibited the highest inhibitory effect against both Gram-negative bacterial strains. Similarly, the inhibitory effect of acetone and acetonitrile against Gram-negative *S. typhi* was almost similar to that of the EtOAc fraction. Oppositely, the EtOH fraction was found to be most effective against Gram-positive strains. Furthermore, the inhibitory effect of the EtOAc fraction against *S. aureus* was almost comparable with the ethanol fraction. Nonpolar fractions of hexane and chloroform were reported to be least effective against all the bacterial strains. This may implicit that the pharmacologically active antimicrobial molecules might be hydrophilic in nature. MIC and MBC of all the fractions (Table 3) showed a proportional correlation with the data obtained from the disc diffusion method. According to a study conducted in Bangladesh, seeds of *M. repandus* have a moderate inhibition effect against *S. typhi*, *B. subtilis*, and *E. coli* [39]. However, there is no study on the antibacterial activity of different fractions and their MIC and MBC. Furthermore, this study has explored the potent inhibitory effect of different fractions against Gram-negative bacteria *S. typhi* for the first time.

The seeds of *M. repandus* are a rich source of antifungal and antitumor maytansinoids such as trewiasine, dehydrotrewiasine, treflorine, trenudine, and N-methyl trewiasine [40, 41]. Besides these, seeds also contain potent antimicrobial compounds such as alkaloids, ricinidine and neolignans, glyceride oil, and pyridinone alkaloids [42]. In a previous study, five neolignans were isolated from the seed endothelium of *M. repandus*, among which 9′-butyl americanol A and americanin showed the antibacterial activity against Gram-positive bacterium *S. aureus* and Gram-negative bacterium *Mycobacterium tuberculosis* [43]. A previous study has reported that neolignans present in *Piper regnellii* have a potent antibacterial activity against *S. aureus* and *B. subtilis* [44].

In a previous study on *P. guajava*, it is revealed that its methanolic extracts showed little antibacterial effect against two Gram-negative bacteria (*E. coli* and *S. enteritidis*) and two Gram-positive bacteria (*S. aureus* and *B. cereus*) [45], but in our study, there was no zone of inhibition. Essential oils obtained from the hydrodistillation of *V. negundo* fresh leaves showed promising antibacterial effects against *B. subtilis* and *E. coli* [46]. According to a previous study on *C. repens*, *S. aureus* was more susceptible to the EtOH extracts of *C. repens* [47]. MeOH extracts of *C. repens* showed a significant antibacterial activity against *S. aureus*, *B. subtilis*, *E. coli*, and *P. aeruginosa* [48]. However, in this study, the methanolic extract of *C. repens* was used, and hence, there was no zone of inhibition observed. The leaves [49] and bark [50] of *A. catechu* showed antibacterial activity against *Streptococcus mitis, Streptococcus sanguis,* and *Lactobacillus acidophilus*, but in this study, we used resin of respective plants, and no zone of inhibition was observed. Similarly, the chloroform extract of *F. religiosa* fruit was found to be ineffective against the bacterial strains used in this study, although it has a moderate effect against other strains such as *S. faecalis, B. megaterium,* and *Azotobacter chrooeoccum* [51]. It has been reported that the chloroform extract of *T. sinensis* flower has an inhibitory effect against *Pseudomonas aeruginosa* [52]. Furthermore, the acetone extract of *E. serratus* fruit was found to be slightly effective against *S. aureus* bacteria [53]. Although the antimicrobial efficacy of *N. nucifera* flower extract has been verified against both Gram-positive and Gram-negative bacteria, the effect of seeds has not yet been justified [54]. Seed oil of *C. sativa* has revealed a measurable zone of inhibition against both Gram-positive and Gram-negative bacteria, but the MeOH seed extract could not exhibit a measurable bacterial inhibitory effect in this study [55]. Moreover, the MeOH extract of *B. utilis* bark has shown a moderate bacterial inhibitory effect in a previous study [56].

Discussing the study's limitations, it is important to note that the potent fractions, specifically the acetone fraction of *Q. infectoria* and the EtOAc fraction of *M. repandus*, could not undergo chromatographic separation to isolate the distinct active compounds. To enhance the standardization of plant extracts and fractions, employing HPLC analysis would be advisable.

## 5. Conclusion

Our study concluded that all the plants used in folk medicine may not have medicinal value; thus, the use of evidence-based traditional medicine is necessary for the rational and effective use of ethnomedicinal plants. Among 15 different plants that were used by traditional healers of Nepal, only the galls of *Q. infectoria* and seeds of *M. repandus* showed significant antibacterial properties against both Gram-positive and Gram-negative bacteria. *Q. infectoria* was reported to be more active against Gram-positive bacteria, whereas *M. repandus* showed more effectiveness against Gram-negative bacteria. The acetone fraction of *Q. infectoria* was more active against Gram-positive bacteria, whereas the ethyl acetate fraction of *M. repandus* was the most active against Gram-negative bacteria.

Based on our research findings, we recommend the utilization of extracts derived from *Q. infectoria* galls and *M. repandus* seeds as potential inhibitors of both Gram-positive and Gram-negative bacteria. In the future, it is essential to conduct bioassay-guided analysis of these plants in order to identify the specific bioactive compounds responsible for their antibacterial activity. In addition, we propose conducting an investigation into the antibacterial properties of all the plants indigenous to Nepal that have been traditionally used for treating various infection-related diseases. This comprehensive study is necessary as not all plants may possess genuine antibacterial effects, and it will help identify and validate the plants with significant antibacterial potential.

## Figures and Tables

**Table 1:** List of selected medicinal plants with their medicinal parts.

S.No.	Biological source	Family	Common name	Parts used	Voucher specimen
1	*Acacia catechu* (L.f.) Wild.	Leguminaceae	Khair	Resin	PUH-072-17
2	*Acacia rugata* (Lam.) Fawc. & Rendle	Leguminaceae	Sikakaai	Fruits	PUH-072-18
3	*Betula utilis* D. Don	Betulaceae	Bhojpatra	Bark	PUH-072-19
4	*Cannabis sativa* L.	Cannabaceae	Bhang	Fruit	PUH-072-20
5	*Cissus repens* Lam.	Vitaceae	Jogeelahara	Climber	PUH-072-21
6	*Drynaria propinqua* (Wall. ex Mett.) Bedd.	Polypodiaceae	Kammari	Rhizome	PUH-072-22
7	*Elaeocarpus sphaericus*	Elaeocarpaceae	Rudrakshya	Fruits	PUH-072-23
8	*Fagopyrum megacarpum* (H. Hara)	Polygonaceae	Bhote Khair	Underground	PUH-072-24
9	*Ficus religiosa* L.	Moraceae	Peepal	Fruits	PUH-072-25
10	*Mallotus repandus* (Willd.) Müll.Arg.	Euphorbiaceae	Rhino-apple,	Seeds	PUH-072-26
11	*Nelumbo nucifera* Gaertn.	Nelumbonaceae	Kamal	Seeds	PUH-072-27
12	*Psidium guajava* L.	Myrtaceae	Amba	Fruits	PUH-072-28
13	*Quercus infectoria* G. Olivier	Fagaceae	Majufal	Galls	PUH-072-29
14	*Tinospora sinensis* (Lour.) Merr.	Menispermaceae	Gujro	Climber	PUH-072-30
15	*Vitex negundo* L.	Lamiaceae	Nirgundi	Aerial	PUH-072-31

**Table 2:** Measurement of the bacterial zone of inhibition shown by fifteen investigated plant samples.

Concentrations (1 mg/disc)	Zone of inhibition (mm)				
Samples		*S. aureus*	*B. subtilis*	*E. coli*	*S. typhi*
*A. catechu*		ND	ND	ND	ND
*A. rugata*		ND	ND	ND	ND
*B. utilis*		ND	ND	ND	ND
*C. sativa*		ND	ND	ND	ND
*C. repens*		ND	ND	ND	ND
*D. propinqua*		ND	ND	ND	ND
*E. sphaericus*		ND	ND	ND	ND
*F. megacarpum*		ND	ND	ND	ND
*F. religiosa*		ND	ND	ND	ND
*M. repandus*		10.00 ± 0.57	13.00 ± 0.57	16.33 ± 0.66	21.66 ± 0.32
*N. nucifera*		ND	ND	ND	ND
*P. guajava*		ND	ND	ND	ND
*Q. infectoria*		30.33 ± 0.32	25.66 ± 0.66	12.00 ± 0.57	14.00 ± 0
*T. sinensis*		ND	ND	ND	ND
*V. negundo*		ND	ND	ND	ND
Amoxicillin		32.33 ± 2.18	17.66 ± 0.32	0	0
Norfloxacin		0	0	11.00 ± 0.66	30.00 ± 0.87

All the values represented mean ± SEM (*n* = 3). ND indicates ZOI are not detected at the examined concentration.

**Table 3:** Minimum inhibitory concentration (MIC) and minimum bactericidal concentration (MBC) for methanolic extract and different fractions of *Q. infectoria* (galls part) and *M. repandus* (seeds part).

S.No.	*Q. infectoria*(galls part)	*S. aureus* (mg/mL)	*B. subtilis* (mg/mL)	*E. coli* (mg/mL)	*S. typhi* (mg/mL)
1	MIC (MeOH extract)	0.31	0.62	2.5	1.25
2	MBC (MeOH extract)	1.25	2.5	5	2.5
3	MIC (EtOAc fraction)	0.23	0.84	2.41	1.24
4	MBC(EtOAc fraction)	1.08	3.34	4.55	2.52
5	MIC (acetone fraction)	0.19	0.85	2.68	1.27
6	MBC (acetone fraction)	0.98	3.39	5.7	2.67
7	MIC (acetonitrile fraction)	0.26	0.82	1.89	0.65
8	MBC (acetonitrile fraction)	1.17	3.32	3.81	1.31
	MIC (EtOH fraction)	0.65	1.29	4.7	2.42
9	MBC (EtOH fraction)	2.57	4.68	9.82	4.83

	*M. repandus* (seeds part)				

10	MIC (MeOH extract)	1.25	1.25	0.62	0.31
11	MBC (MeOH extract)	5	2.5	1.25	0.62
12	MIC (hexane fraction)	1.29	1.28	1.79	0.63
13	MBC (hexane fraction)	5.4	2.6	3.58	1.25
14	MIC (chloroform fraction)	1.27	2.4	1.28	0.58
15	MBC (chloroform fraction)	5.21	5.1	2.61	1.20
16	MIC (EtOAc fraction)	0.62	1.24	0.48	0.25
17	MBC(EtOAc fraction)	2.43	2.54	0.99	0.53
18	MIC (acetone fraction)	0.65	1.25	0.52	0.29
19	MBC (acetone fraction)	2.7	2.57	1.12	0.59
20	MIC (acetonitrile fraction)	1.29	1.35	0.64	0.28
21	MBC (acetonitrile fraction)	5.4	2.8	1.29	0.57
22	MIC (EtOH fraction)	0.59	1.22	0.82	0.45
23	MBC (EtOH fraction)	2.35	2.54	1.84	0.96

## Data Availability

The data used to verify the results of this investigation are included within the article.

## Supplementary Materials

Supplementary Materials The diagrams of the zone of inhibition shown by different extracts are available in Supplementary Figure S1.
