# Supplementary material for: Preliminary Study on the Antibacterial Activities and Antibacterial Guided Fractionation of Some Common Medicinal Plants Practices in Itum Bahal, Kathmandu Valley of Nepal
Source: ScientificWorldJournal. 2023 Sep 21;2023:7398866. doi: 10.1155/2023/7398866 (PMC10539093; doi:10.1155/2023/7398866)
Supplement: Supplementary Materials — The diagrams of the zone of inhibition shown by different extracts are available in Supplementary Figure S1. [file 7398866.f1.docx]

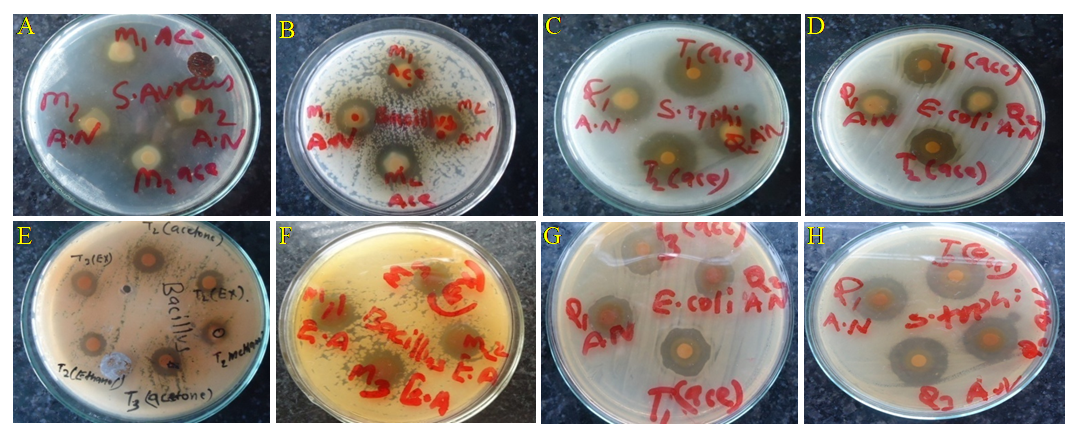


Figure S1. ZOI inhibition of acetone and acetonitrile fraction of *Q. infectoria*a gainst different bacterial strains (A: *S.* a*ureus*, B: *B. subtilis*, C: *S. typhi*, D: *E. coli* ) along with ZOI inhibition of different fractions of *M. repanus* against different bacterial strains (E: *E. coli*, F: *B. subtilis*, G: *E. coli* for ethyl acetate fraction, H: *S. typhi* for acetone and acetonitrile fraction)

d

c

a
